# Effect of lactation therapy on *Staphylococcus aureus* transmission dynamics in two commercial dairy herds

**DOI:** 10.1186/1746-6148-9-28

**Published:** 2013-02-11

**Authors:** John W Barlow, Ruth N Zadoks, Ynte H Schukken

**Affiliations:** 1 Department of Animal Science, University of Vermont, Burlington, VT 05401, USA; 2 Moredun Research Institute, Pentlands Science Park, Bush Loan, Penicuik, Scotland, EH26 0PZ, UK; 3 Quality Milk Production Services, Department of Population Medicine and Diagnostic Sciences, Cornell University, Ithaca, NY, 14853, USA

**Keywords:** Mastitis, Lactation therapy, Molecular diagnostics, *Staphylococcus aureus*

## Abstract

**Background:**

Treatment of subclinical mastitis during lactation can have both direct (individual animal level) and indirect (population level) effects. With a few exceptions, prior research has focused on evaluating the direct effects of mastitis treatment, and to date no controlled field trials have been conducted to test whether beneficial indirect effects of lactation treatment strategies targeting subclinical mastitis can be demonstrated on commercial dairy farms. Furthermore, there is limited knowledge on the impact of such interventions on the population dynamics of specific bacterial strains. The purpose of this study was to test the hypothesis that lactation therapy targeting *S. aureus* subclinical intramammary infection reduces transmission of *S. aureus* strains within dairy herds. Pulsed-field gel electrophoresis (PFGE) and multilocus sequence typing (MLST) were used to determine strain specific infection dynamics in treated and control groups in a split herd trial conducted on 2 commercial dairy farms.

**Results:**

The direct effect of 8 days intramammary lactation therapy with pirlimycin hydrochloride was demonstrated by an increased proportion of cure and a reduction in duration of infection in quarters receiving treatment compared to untreated controls. The indirect effect of lactation therapy was demonstrated by reduction of new *S. aureus* intramammary infections (IMI) caused by the dominant strain type in both herds. Strain typing of representative isolates taken over the duration of all IMI, including pre- and post-treatment isolates, provided more precise estimates of new infection, cure, and re-infection rates. New *S. aureus* infections in recovered susceptible quarters and the emergence of a new strain type in one herd influenced incidence measures.

**Conclusion:**

In addition to demonstrating positive direct effects of lactation therapy, this study provides evidence that treatment of subclinical *S. aureus* mastitis during lactation can have indirect effects including preventing new IMI and reducing incidence of clinical mastitis within dairy herds. Strain specific transmission parameter estimates for *S. aureus* MLST clonal complexes 5, 97 and 705 in 2 commercial dairy herds are also reported.

## Background

Mastitis continues to be recognized as one of the most economically important health problems of dairy cattle [1,2]. Subclinical mastitis, which can be characterized by an elevated milk somatic cell count (SCC), is the dominant form affecting cows. A number of authors have suggested that dairy producers frequently leave subclinical mastitis undetected or untreated for extended periods during lactation [3-5].

Mastitis control interventions are intended to reduce the duration of infection and prevent new infections. Examples of effective mastitis control practices include the use of long acting antibiotics at the end of lactation (dry-cow therapy), application of a post-milking teat disinfectant following each milking, and segregation or culling of infected individuals [6,7]. Treatment of mastitis during lactation has been predominately limited to the treatment of clinical cases, although the value of treating subclinical mastitis during lactation is well recognized as a component of *Streptococcus agalactiae* control [8]. The potential impact of lactation therapy on transmission of other major gram-positive mastitis pathogens (e.g. *Staphylococcus aureus* and *Streptococcus uberis*) has been estimated using a mathematical model which accounted for both direct and indirect effects of treatment [9]. Previous field studies have examined the direct effect of lactation therapy for subclinical mastitis [10,11]. Delaying successful treatment of subclinical *S. aureus* IMI may increase the duration of infection, reduce the probability of cure and increase the risk of exposure for uninfected quarters [12,13]. Therefore, successfully treating cases of subclinical mastitis has a direct effect on the treated animal, but may also have an indirect effect by reducing new infection risk in uninfected animals in the herd [9,13,14]. To date no controlled field trials have been conducted to test whether the predicted beneficial indirect effects of subclinical mastitis lactation treatment strategies can be demonstrated on commercial dairy farms. Furthermore, there is limited knowledge on the impact of such interventions on the population dynamics of specific bacterial strains. For example, *S. aureus* strains may be identified using molecular methods including pulsed-field gel electrophoresis (PFGE) and multilocus sequence typing (MLST), and strain specific differences in clinical manifestations, host-adaptation, and response to therapy have been identified [13,15,16].

In this report we describe the results of a field trial that evaluated a diagnosis driven treatment program targeting subclinical *S. aureus* IMI and applied PFGE and MLST to identify strain specific *S. aureus* infection dynamics. Outcomes evaluated in this study included cure proportions, duration of infection, infection prevalence and incidence, and rates of clinical mastitis and mastitis associated culling.

## Methods

### Study design

A negative-controlled treatment trial was conducted for a period of 13 months on 2 commercial dairy herds (one each in New York and Vermont, USA) milking Holstein dairy cows 3 times per day in a milking parlor (Table 1). Criteria for herd participation included: 1) reliable individual cattle identification; 2) housing of lactating dairy cattle in two or more comparable groups (“strings”) of approximately 100 cows in separate pens (free-stall housing); 3) enrolled in a Dairy Herd Improvement Association (DHIA) monthly testing program including SCC; 4) an average monthly herd SCC between 250,000 and 500,000 cells/ml; 5) accepted mastitis control practices applied to all cows, including use of pre- and post-milking teat disinfectants, and blanket use of dry-cow therapy; 6) segregated housing for lactating cows receiving antibiotic treatments; 7) owner willing to keep records on all cows, including dates of calving, entries and exits from lactating cow pen groups, clinical disease, treatment, and culling. Herd owners were compensated for participation in the study. The study was conducted with approval of the Cornell University and University of Vermont Institutional Animal Care and Use Committees (IACUC).

**Table 1:** **Production, SCC, infection status, and *Staphylococcus aureus *isolation events for treatment and control group**

**Herd**	**Pen**	**Monthly average number lactating cows**	**Monthly average milk production (kg)**	**Monthly average SCC (cells/ml x 1000)**	**Total Quarter Days at risk for new IMI**^**1**^	**Total Quarter Days Infected**^**2**^	**Number *S. aureus *isolates (# quarters/# cows)**	
							**IMI associated isolates**^**3**^	**incidental isolates**^**3**^
1	treatment	95	31.4	419	129984	1335	80(21/20)	15(13/11)
	control	98	32.7	401	130254	2479	137(25/21)	22(20/17)
	whole herd^4^	319	32.7	404			221(50/45)	37(33/28)
2	treatment	91	42.3	292	125241	1345	58(11/9)	2(2/2)
	control	89	41.4	296	116295	14	1(1/1)	0
	whole herd	346	35.0	298			68(21/16)	4(4/4)

^1 ^Sum of individual quarter days uninfected at risk for new intramammary infection (IMI).

^2 ^Sum of individual quarter days infected based on midpoint estimates of IMI start and stop dates [17].

^3 ^IMI status based on definitions of Zadoks et al. [12]; incidental isolates are those not meeting IMI criteria.

^4 ^Whole herd totals include all lactating cows during study period, (cows that entered treatment and control groups, plus cows never housed with these groups); number of isolates obtained from whole herd exceeds sum of treatment and control pens as 13 IMI isolates and 2 incidental isolates were obtained from milk samples collected from cows sampled at the start of a lactation and either the IMI cured prior to entry into a study pen or the cow did not enter a study pen during the course of the trial.

Treatment was randomly allocated to one of 2 pens within each herd and the treatment unit was a group (pen) of lactating cows. In the month preceding the start of the study, cows were systematically assigned to pens based on existing odd or even unique identification numbers. Cows that calved during the study were assigned to either the treatment or control pens based on their odd-even identification number. Using this randomization method no differences in mean parity, days in milk, or SCC, were found among treatment and control groups within herds at the start of the study. Groups within herds were a dynamic population, with entries and exits of individuals into the study population following normal management cycles. Dates of all entries and exits to treatment or control pens were recorded for each cow. Pens of cows within each herd were milked in the following order at each milking session: fresh cow group (cows < 30 days in milk), study treatment group, study control group, additional pens not enrolled in study. The milking system was washed and sanitized 3 times daily between each milking of the entire herd.

### Milk sample collection and bacteriologic analysis

Composite milk samples were collected monthly by DHIA technicians and processed through regional commercial testing laboratories for SCC testing. Individual quarter milk samples were collected for microbiologic analysis from all cows in control and treatment groups at the start of the study, at monthly intervals for the duration of the study, and at the end of the study. These quarter samples were collected within 3 days of DHIA monthly composite sample collection. Quarter milk samples were also collected from all cows within 3 days following parturition (fresh sample), immediately following identification of clinical mastitis (clinical pre-treatment sample), immediately prior to treatment of subclinical mastitis (subclinical pre-treatment sample), at any time when cows were added to or removed from the study pens for greater than 24 hours (entry/exit sample), immediately prior to exit from the herd (cull sample), and at approximately 7, 14, 21, and 28 days following cessation of any antibiotic therapy (clinical or subclinical post-treatment samples). Monthly sample collection was conducted by trained field technicians, while farm personnel were trained to collect all additional samples using aseptic methods [18]. All samples were stored frozen for up to 2 weeks prior to being thawed over-night under refrigeration for aerobic bacteriologic culture (10 uL of each milk sample plated on tryptic soy agar with 5% sheep’s blood and 1% esculin plate and incubated for up to 48 hours at 37°C). Interpretation of culture results was performed according to established guidelines [18]. Samples with ≥3 morphologically distinct colony types were considered contaminated and eliminated from analysis.

### Infection status

Sequential SCC measurements in combination with bacteriologic culture were used to identify cows with subclinical IMI and eligible for lactation therapy. This procedure was intended to model a practical culture-based diagnostic program that might be applied on commercial farms where an elevated composite cow-level SCC is used to trigger quarter-level bacteriologic culture and culture results are used to trigger a decision to intervene (for example treat, segregate or cull the infected quarter or cow). Sequential cow level SCC was evaluated to identify cases of subclinical mastitis which were defined as composite milk SCC ≥ 200,000 cells/ml for 2 or more serial monthly samples. Sequential quarter level bacteriologic culture was used to define IMI status. Throughout this report, we use the term “subclinical mastitis” to describe cases of mastitis defined by SCC measures, and “IMI” or “subclinical IMI” to describe cases of mastitis defined by bacteriologic culture. An individual quarter was defined as having a subclinical IMI, when having no occurrence of clinical mastitis within the previous 14 days and meeting the criteria of Zadoks et al. [12] (i.e. ≥ 1000 CFU/ml of *S. aureus* cultured from a single sample, ≥ 500 CFU/ml of *S. aureus* cultured from two out of three consecutive samples, or ≥100 CFU/ml of *S. aureus* cultured from three consecutive samples). Isolations of *S. aureus* from uncontaminated samples that did not meet these criteria were defined as incidental isolation events. Cure of a subclinical IMI following therapy was defined when a quarter was culture negative for the pre-treatment species or strain on 4 of 4 weekly post treatment samples. An untreated quarter with a subclinical IMI was defined as spontaneously cured when the quarter was negative for the same species or strain on 2 consecutive samples taken at least one month apart. Clinical mastitis was defined as an abnormality in appearance or consistency of milk, with or without localized (e.g. swollen quarter) or systemic signs. A clinical IMI was defined as a case of clinical mastitis with a *S. aureus* positive bacteriologic culture (i.e. ≥100 CFU/ml). A new clinical mastitis event was identified at the quarter level after that quarter was observed free of clinical signs for ≥ 14 days or if clinical mastitis occurred within 14 days of a previous case but was caused by a different bacterial species or strain. A clinical IMI was defined as a bacteriologically cured when culture negative for the pre-treatment species or strain on 4 of 4 weekly post treatment samples. These definitions recognize that quarters defined as having a subclinical IMI could have sporadic clinical events, either presenting as clinical flare-ups during a subclinical IMI or as an initial clinical event which subsequently becomes a subclinical case [12,14].

### Treatment program

The objective of the diagnostic program was to target lactation therapy to cows with subclinical *S. aureus* IMI. Cows assigned to the treatment group were eligible for lactation therapy if they had an elevated composite SCC (≥200,000 cells/ml) in the current month, plus an elevated SCC (≥200,000 cells/ml) in one of the two previous months in combination with a *S. aureus* positive culture result in at least one quarter in the current month. All lactating quarters of eligible cows were treated, regardless of the number of infected quarters, because there is a within cow correlation of infection risk among quarters [19]. Treatment of all 4 quarters in infected cows thus decreased the potential effect of within cow interdependence of quarters on transmission and incidence estimates. This cow level (“blanket”) intramammary treatment during lactation was not intended to model a recommended practice for adoption by commercial dairy producers. Treatment was with a commercially available intramammary formulation of pirlimycin hydrochloride (Pirsue™, Pfizer Animal Health, New York, USA) at the labeled dosage (50 mg) once daily for 8 days [10]. Cows diagnosed with subclinical IMI in the control group received no therapy. Cows experiencing clinical mastitis in either group were treated using the established practices of the participating farms and records were maintained on the type and duration of therapy. During a 3-month ‘pre-intervention’ observation period at the start of the study there was no treatment of subclinical mastitis in the treatment group. All data were collected during both the initial 3 month observation and the subsequent 10 month intervention periods.

### Species identification and *S. aureus* strain typing

Presumptive *Staphylococcus* spp. colonies were identified based on growth characteristics, and were transferred to a blood agar plate for isolation and further identification. All catalase-positive, hemolytic, gram-positive cocci were tested for coagulase activity by the tube coagulase test [18]. Coagulase-positive gram-positive cocci were transferred to broth culture and stored at −80°C on ceramic beads (CryoBank beads, Copan Diagnostics Inc., Murrieta, CA) in tryptone soy broth with 15% glycerol. All stored coagulase-positive staphylococci were tested by PCR amplification of the *S. aureus* specific thermonuclease gene (*nuc*) using established methods to confirm species identity [20]. Within each IMI, representative *S. aureus* isolates were selected from storage for strain typing by PFGE and MLST. In quarters having more than one isolation event over time, isolates were selected for strain typing from early (e.g. the first isolation event), middle (one or more subsequent isolation events), and late (one of the final isolation events) time points. In addition, pre- and post-treatment isolates were selected for strain typing from all cows receiving lactation therapy.

PFGE typing was conducted as described by McDougal et al. [21]. PFGE pulsotypes (PTs) were identified by visual examination of gels by two independent observers (JWB and RNZ) using established criteria [22] with different types identified by > 3 band differences on restriction digest patterns. Isolates that differed by 1 to 3 bands were defined as a subtype. MLST was conducted as described by Enright et al. [23] with alleles, sequence types (ST), and clonal complexes (CC) assigned using the MLST database. Novel alleles and STs (allelic profiles) were assigned designations by the database curator following submission of DNA sequence trace chromatograms (http://www.mlst.net). Clonal complexes and new STs were defined as bovine- or human-adapted using methods described by van den Borne et al. [16].

Susceptibility testing for the antimicrobials ampicillin, cephalothin, ceftiofur, erythromycin, oxacillin, pirlimycin, penicillin, penicillin/novobiocin, and tetracycline was conducted on isolates selected for strain typing using broth micro-dilution minimum inhibitory concentration methods (MIC; Sensititre, Trek Diagnostic Systems, Cleveland, Ohio, USA) following Clinical Laboratory Standards Institute (CLSI) standards [24]. MIC_50_ and MIC_90_ values were calculated for any PT or ST with 3 or more IMI using the methods of Schwarz et al.[25]. Presence of genetic markers associated with penicillin resistance (*blaZ*) and methicillin resistance (*mecA*) were tested by PCR using established methods [26,27] for all isolates selected for strain typing, and these isolates were also screened for beta-lactamase expression using a nitrocefin assay [28].

### Duration of infection

Duration of infection was calculated based on the mid-point estimation method previously described by Zadoks et al. [17]. Infected quarters entering the study, either at the start of the study or at the start of a lactation, were considered left censored and the IMI start date was the date of first positive culture. Infected quarters exiting the study were considered right censored and the IMI end date was the date of exit or the end date of the study. Quarter days infected at pen or herd level (i.e. the sum of IMI prevalence days for individual quarters of all animals) were calculated based on recorded dates of cow entry and exit from the study pens for all lactating quarters. Quarter days susceptible (i.e. days at risk for new IMI among uninfected quarters) were calculated based on recorded dates of cow entry and exit from the study pens accounting for the date of change in quarter infection status during each interval. Non-lactating quarters in lactating cows were identified with the date of milking cessation of the individual quarters (i.e. quarter ‘culling’) so that any ‘blind’ or culled quarters within lactating cows did not contribute to the number of infected or susceptible quarter days in the pen or herd.

### Statistical methods

All statistical analysis was conducted using SAS version 9 (SAS Institute, Inc., Cary, NC, USA). Difference in the proportion of quarters cured in treatment groups was tested using Fisher’s exact test. Pearson chi square or Fisher’s exact test were used to test for associations between treatment group and the proportion of quarters becoming re-infected following cure, or the proportion of quarters with at least one clinical mastitis event, or the proportion of quarters culled due to mastitis. The proportion of *S. aureus* clinical mastitis events was analyzed using two measures, either as a count of quarters with at least one clinical mastitis event to eliminate potential bias associated with repeated events within a quarter, or as the total count of clinical episodes. The hypothesis that treatment affected duration of infection was tested using the Log-rank test to compare the Kaplan-Meier survival function for *S. aureus* IMI in treatment and control groups, while controlling for the effect of PT or ST. We also modeled the effect of treatment on duration of infection using a linear regression model including PT or ST as a covariate.

Prevalence of *S. aureus* IMI (number of quarters days infected, *P*_*I*_ ), and incidence of new *S. aureus* IMI (number of new IMI, *I*_*N*_ ), were analyzed as outcomes using generalized linear models (PROC GENMOD, SAS) and model checking included examination of deviance and Pearson Chi-square for goodness of fit [12,29]. Evidence of overdispersion was adjusted using Pearson Chi-square estimates divided by the degrees of freedom (Pscale option). Binomial, Poisson, or Negative Binomial error distributions were selected for final models after comparisons for goodness of fit. Backward elimination of variables was used to select the final models with treatment group (*Group*), the primary effect of interest, forced into all final models. Additional independent variables and their 2- and 3-way interactions where included when *p*<0.10. Additional variables included in the full models were farm, time period (*Timep*), and PT or ST when prevalence was the dependent variable, and these same variables plus prevalence (*P*_*I*_) when incidence was the dependent variable. Time period was modeled with 2 categories defining the pre-intervention (observation months 1–3) period as 0 and the intervention period (months 4–13) as 1. Because we observed differences in strain dynamics over time during the intervention period, we also modeled time period with 3 categories, by separating pre-intervention (*pre*, months 1–3), early intervention (*earlytp*, months 4–8) and late intervention (*latetp*, months 9–13) periods. In the final prevalence model, the impact of treatment group on the prevalence in each interval was modeled assuming a negative binomial error distribution, a log link, and an offset of the total number of quarter days,

(1) $$\varepsilon \left[\ln\left(P_{I}\right)\right]=\text{intcpt}+\text{Group}+\text{Timep}+\text{Grou}p^{*}\text{Timep}+\ln\left(N\right)$$

where *ε* = expected value and ln(*N*)= offset. Comparisons of least square means IMI quarter-days prevalence (*P*_*I*_) between treatment and control groups and across time periods was conducted separately for each farm using model 1.1 and accounting for multiple comparisons with Bonferroni methods.

The impact of treatment on incidence in each interval was estimated in a Poisson regression model, with a Poisson error distribution, a log link, and an offset of the number of quarter days susceptible for new IMI (*S*),

(2) $$\varepsilon \left[\ln\left(I_{N}\right)\right]=\text{intcpt}+\text{Group}+\text{Timep}+\text{Grou}p^{*}\text{Timep}+\ln\left(S\right)$$

Since prevalence was affected by treatment group (covariance estimate = 0.46, p=0.018), prevalence was not included as a covariate in model 1.2.

Transmission parameters (*β*_*i*_) were estimated from a generalized linear model with number of new IMI events in each monthly interval (*I*_*N*_) as the outcome, a log link, assuming a Poisson error distribution, and offset ln (S*I/N) [12,29],

(3) $$\varepsilon \left[\ln\left(I_{N}\right)\right]=\beta_{i}^{*}+\ln\left(S\times \frac{I}{N}\right)$$

where β*= ln(*β*), *S* = quarter days susceptible, *I* = quarter days infected, and *N* = total quarter days in each interval. Separate transmission parameters (including 95% confidence intervals) were estimated for the individual farms, as well as between treatment periods, groups, and for STs. The univariate effects of farm, treatment group, time period, and ST on transmission parameter estimates were estimated by inclusion of these variables in model 1.3.

## Results

### Descriptive analysis

A total of 31,761 quarter milk samples were collected from the two herds, with *S. aureus* isolated from 330 samples. From Herd 1, there were 14,467 quarter samples taken from 3675 sample events from 385 cows, with 209 (1.4%) samples identified as contaminated leaving 14,258 quarter samples for analysis. Of these, *S. aureus* was isolated from 258 (1.8%) samples. From Herd 2, there were 17,294 quarter samples taken from 4387 sample events from 580 cows, with 787 (4.6%) samples identified as contaminated leaving 16,507 quarter samples for analysis. Of these, *S. aureus* was isolated from 72 (0.4%) samples. *S. aureus* was not identified in any samples classified as contaminated from either herd. Table 1 summarizes the herd production, SCC, and *S. aureus* IMI data by treatment group within herd.

### Strain typing

Nine PTs were recognized to cause *S. aureus* IMIs, with up to 3 subtypes identified within 4 of the PTs (Table 2). Twelve STs were identified in these herds including STs in bovine host-associated CCs 97, 479, and 705, and human host-associated STs 8, 20, 25, and 87. Four previously unreported alleles and 3 new STs were identified and clustered within CCs 97 and 705 (Table 2).

**Table 2:** **Strain typing of 171 selected *Staphylococcus aureus *isolates from 2 herds**^**1**^

**Strain type**			**IMI**^**5**^	**Herd**	**In vitro Antimicrobial Susceptibility-Minimum Inhibitory Concentration values, MIC**_**50**_**/MIC**_**90 **_**(ug/ml)**^**6**^									**antibiotic resistance PCR *blaZ/mecA***
**PT**^**2**^	**ST**^**3**^	**MLST CC**^**4**^			**Amp**	**Ceph**	**Ceftio**	**Ery**	**Oxa**	**Pen**	**Pen: Novo**	**Pirl**	**Tet**	
1	151	705	3	2	≤0.12/0.25	≤2/≤2	≤0.5/1	≤0.25/≤0.25	≤2/≤2	≤0.12/≤0.12	≤(1:2)/≤(1:2)	≤0.5/1	≤1/≤1	−/−
	2185	705	22	1	≤0.12/0.25	≤2/≤2	≤0.5/4	≤0.25/≤0.5	≤2/≤2	≤0.12/≤0.12	≤(1:2)/≤(1:2)	≤0.5/≤0.5	≤1/2	−/−
1a	2185	705	1	1	≤0.12	≤2	1	≤0.25	≤2	≤0.12	≤(1:2)	≤0.5	≤1	−/−
1b	2185	705	6	1	≤0.12/≤0.12	≤2/≤2	≤0.5/2	≤0.25/≤0.25	≤2/≤2	≤0.12/≤0.12	≤(1:2)/≤(1:2)	≤0.5/≤0.5	≤1/≤1	−/−
	2185	705	3	1	≤0.12/≤0.12	≤2/≤2	1/1	≤0.25/≤0.25	≤2/≤2	≤0.12/≤0.12	≤(1:2)/≤(1:2)	≤0.5/≤0.5	≤1/≤1	−/−
1c	nd		1	1	≤0.12	≤2	1	≤0.25	≤2	≤0.12	≤(1:2)	≤0.5	4	−/−
2	2189	97	5	1	≤0.12/≤0.12	≤2/8	1/1	≤0.25/1	≤2/≤2	≤0.12/≤0.12	≤(1:2)/≤(1:2)	≤0.5/1	≤1/2	−/−
	2189	97	7	1	≤0.12/≤0.12	≤2/≤2	1/1	≤0.25/≤0.25	≤2/≤2	≤0.12/≤0.12	≤(1:2)/≤(1:2)	≤0.5/1	≤1/≤1	−/−
2a	nd		1	1	≤0.12	≤2	2	≤0.25	≤2	≤0.12	≤(1:2)	2	4	−/−
2b	nd		4	1	≤0.12/≤0.12	≤2/≤2	1/1	≤0.25//≤0.25	≤2/≤2	≤0.12/≤0.12	≤(1:2)/≤(1:2)	≤0.5/2	2/4	−/−
3	8	5	7	2	0.5/0.5	≤2/≤2	1/1	≤0.25/≤0.25	≤2/≤2	0.25/0.5	≤(1:2)/≤(1:2)	≤0.5/≤0.5	≤1/≤1	+/−
4	352	97	2	2	≤0.12	≤2	2	≤0.25	≤2	≤0.12	≤(1:2)	≤0.5	≤1/≤1	−/−
4a	352	97	5	2	≤0.12/≤0.12	≤2/4	1/1	0.5/0.5	≤2/≤2	≤0.12/≤0.12	≤(1:2)/≤(1:2)	≤0.5/≤0.5	≤1/≤1	−/−
4b^fh^	nd		1	1	≤0.12	≤2	1	0.5	≤2	≤0.12	≤(1:2)	≤0.5	≤1/≤1	−/−
5	2187	97	1	1	≤0.12	≤2	1	≤0.25	≤2	≤0.12	≤(1:2)	≤0.5	≤1	−/−
5a	nd		1	1	≤0.12	≤2	1	≤0.25	≤2	≤0.12	≤(1:2)	≤0.5	≤1	−/−
6	nd		1	1	≤0.12	≤2	≤0.5	≤0.25	≤2	≤0.12	≤(1:2)	≤0.5	≤1	−/−
7	20	20	1	2	≤0.12	≤2	≤0.5	≤0.25	≤2	0.25	≤(1:2)	≤0.5	≤1	−/−
8	479	479	1	1	≤0.12	≤2	≤0.5	≤0.25	≤2	≤0.12	2/4	≤0.5	≤1	−/−
9^†^	87	59	0	1	8	4	2	8	4	8	≤(1:2)	1	2	+/+
10	25	15	1	3	16	≤2	1	≤0.25	≤2	8	≤(1:2)	≤0.5	≤1	+/−
11^†^	nd		0	2	≤0.25	≤2	≤0.5	≤0.25	≤2	0.25	≤(1:2)	≤0.5	≤1	−/−

^1 ^Isolates from 74 IMI including 61 IMI identified in cows in treatment and control pens and13 IMI identified in cows that did not enter study pens. An additional 10 incidental isolates were also typed.

^2 ^PFGE pulsotypes; types with the same number but different letters are minor variants with 1–3 band differences.

^3 ^Sequence type determined by MLST.

^4 ^MLST clonal complex predicted founder determined by eBurst MLST database (last searched May 30, 2012).

^5 ^Number of quarters with IMI for each strain type.

^6 ^Antimicrobial susceptibility for ampicillin (Amp), cephalothin (Ceph), ceftiofur (Ceftio), erythromycin (Ery), oxacillin (Oxa), penicillin (Pen), penicillin:novobiocin (Pen:Novo), pirlimycin (Pirl), and tetracycline (Tet); MIC_50 _and MIC_90 _values calculated as described in [25].

† type found as only an incidental isolate, not defined as IMI or associated with subclinical mastitis (SCC< 200,000 cells/ml).

^fh ^type found in fresh heifer with clinical mastitis, cow did not enter study pens.

nd = not determined.

Infection due to a single strain or subtype over time was common. Serial sampling showed a single strain to be associated with 37 of 38 IMI. Strain typing was completed on 2 to 4 (mean =2.1) pretreatment isolates obtained from quarters of cows in the treated groups and 2 to 6 (mean = 3.6) isolates from infected quarters of cows in the control group. Strain typing identified 3 quarters where a difference in the strain was observed between pre-treatment and post-treatment isolates. These quarters were culture negative for 2 and 3 weeks post treatment before a new *S. aureus* strain was isolated.

There was 100% agreement between phenotypic and genotypic test results for penicillin and oxacillin susceptibility. Antimicrobial resistance factors were associated with strain types. ST 8 and 25 were positive for *blaZ* gene PCR and showed increased ampicillin and penicillin MICs, and ST 87 was positive for *blaZ* and *mecA* gene PCR and had increased ampicillin, penicillin, oxacillin and erythromycin MICs, compared to all other strain types which had relatively low MIC values and were *blaZ* and *mecA* gene PCR negative (Table 2).

### Bacteriologic cure and the effect of lactation therapy on clinical mastitis and culling

Table 3 summarizes results of subclinical IMI treated for 8 days with pirlimycin. Twenty-three (38%) of the 61 IMI identified in the study pens were not eligible for 8-day therapy. Reasons that IMI were not eligible for treatment included short duration subclinical IMI not defined as subclinical mastitis based on SCC measures (n=2), IMI identified in the pre-intervention period that either spontaneously cured (n=3) or were right censored during the pre-intervention period (n=6), and clinical IMI events that either cured (n=4) or were culled or dried-off prior to being defined as subclinical IMI (n=8). Re-infections with *S. aureus* following cure were observed for 3 quarters. There was a significant difference in the proportion of quarters cured in the treated groups compared to control groups using data from only Herd 1 or pooled from the 2 herds (p<0.001, Fischer exact test, exact confidence limits undefined; Table 3). In Herd 1 the 10 subclinical IMI that cured following 8-day therapy were caused by CC97 and 705 and in herd 2 the 4 subclinical IMI that cured were all caused by ST8. Most (3 of 4) IMI defined as non-cured IMIs were observed in herd 1 and caused by ST2185 (CC705).

**Table 3:** ***Staphylococcus aureus *IMI eligible for 8-day lactation therapy in treatment and control pens**

**Treatment Pen**									
	**All**	**Subclinical treated**	**Cure**^**1 **^**following subclinical therapy**	**Right censor reason**^**2**^				**Quarter with Clinical events**	
				**exit**	**dry**	**cull**	**Study end**	**IMI started as clinical case**	**Flare-up during existing IMI**
Farm 1	13	13	10	0	0	1	2	1	1
Farm 2	9^*^	5	4	3	1	1	0	0	0
Total	22	18	14	3	1	2	2	1	1
**Control Pen**									
	**All**	**Subclinical treated**	**Spontaneous cure**^**3**^	**Right censor reason**^**2**^				**Quarter with Clinical events**	
				**exit**	**dry**	**cull**	**Study end**	**IMI started as clinical case**	**Flare-up during existing IMI**
Farm 1	16	nt^4^	0	0	6	1	9	3	8
Farm 2	0	nt	0	0	0	0	0	0	0
Total	16	nt	0	0	6	1	9	3	8

^1 ^Bacteriologic cure following treatment of subclinical or clinical IMI is based on negative bacteriologic culture for the pre-treatment Pulsotype (PT) on 4 of 4 samples taken at approximately 7, 14, 21, and 28 days post-treatment.

^2 ^Right censored IMI are classified to include all IMI that are not cured prior to either exit from a study pen to another lactating group (exit), dry-off (dry), culling (cull), or at the conclusion of the study (study end).

^3 ^Spontaneous cure of an IMI is based on 2 sequential negative monthly cultures for the prior PT.

^4 ^nt = not treated, i.e. subclinical IMI that would have been eligible for therapy in control pens but were not treated.

^* ^4 quarters in the treatment group were eligible for subclinical therapy but exited the study pen prior to initiation of therapy (3 exited to another pen and 1 dry-off).

In Herd 1, the odds of a subclinical *S. aureus* IMI displaying a clinical mastitis flare-up in the control group were 22 times greater than in the treatment group (p<0.001, Fischer exact test, exact confidence limits 3 <OR<239). The odds ratio was an unbiased estimate based on data in Table 3, which are the number of quarters with at least one clinical event ignoring repeated clinical episodes within a quarter. However, 3 quarters in the control group displayed recurrent clinical episodes, therefore the total number of clinical events observed were 2 among 13 *S. aureus* IMI in the treatment group compared to 15 among 16 *S. aureus* IMI in the control group. No clinical flare-ups of *S. aureus* mastitis were observed in association with subclinical IMI in Herd 2, however there were 4 clinical *S. aureus* IMI events in post-partum cows that occurred while they were housed in fresh groups (i.e. before they entered the treatment or control groups). These clinical cases were caused by ST151 and ST352. In Herd 1, there were 7 clinical *S. aureus* events not associated with subclinical IMI, and all of these were caused by ST2185.

There were fewer total right censored *S. aureus* IMI in the treatment groups compared to the control groups but there was no difference between groups in the proportion of mastitis associated culling events (Table 3).

### Direct effect of lactation therapy on duration of infection

**Figure 1:**
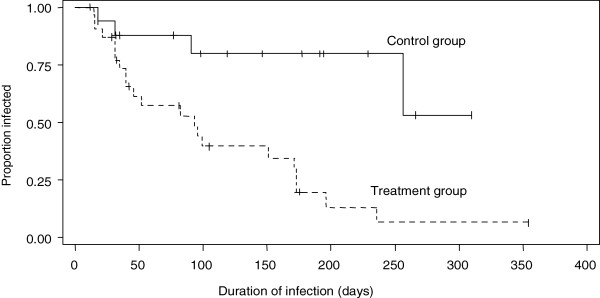
**Kaplan-Meier survival function of subclinical staphylococcus aureus IMI from 2 commercial dairy herds. **The median duration of infection for 22 quarters in the treatment groups (dashed line) estimated to be 93 days was less than the 256 day median duration of infection estimate for 16 quarters in the control groups (solid line) demonstrating a reduction (log rank test p=0.0025) in duration of infection in the groups of cows receiving the intervention.

The arithmetic mean duration of subclinical IMI calculated using the midpoint estimation method was 135 and 86 days for the control and treatment groups respectively. Least square mean estimates of duration from a generalized linear model accounting for the effect of treatment group, *S. aureus* strain, and the interaction between treatment and strain, were similar to the values obtained using the mid-point estimation method, and the effect of treatment was significant (p=0.0072). In a Kaplan-Meier estimate of the survival function of subclinical IMI accounting for censored observations, the median duration of infection was estimated to be 256 and 93 days in the control (n=16) and treatment (n=22) groups respectively, demonstrating a significant reduction in duration of infection in the groups of cows receiving the intervention (log rank test p=0.0025, Figure 1).

### Effect of lactation therapy on IMI prevalence

**Figure 2:**
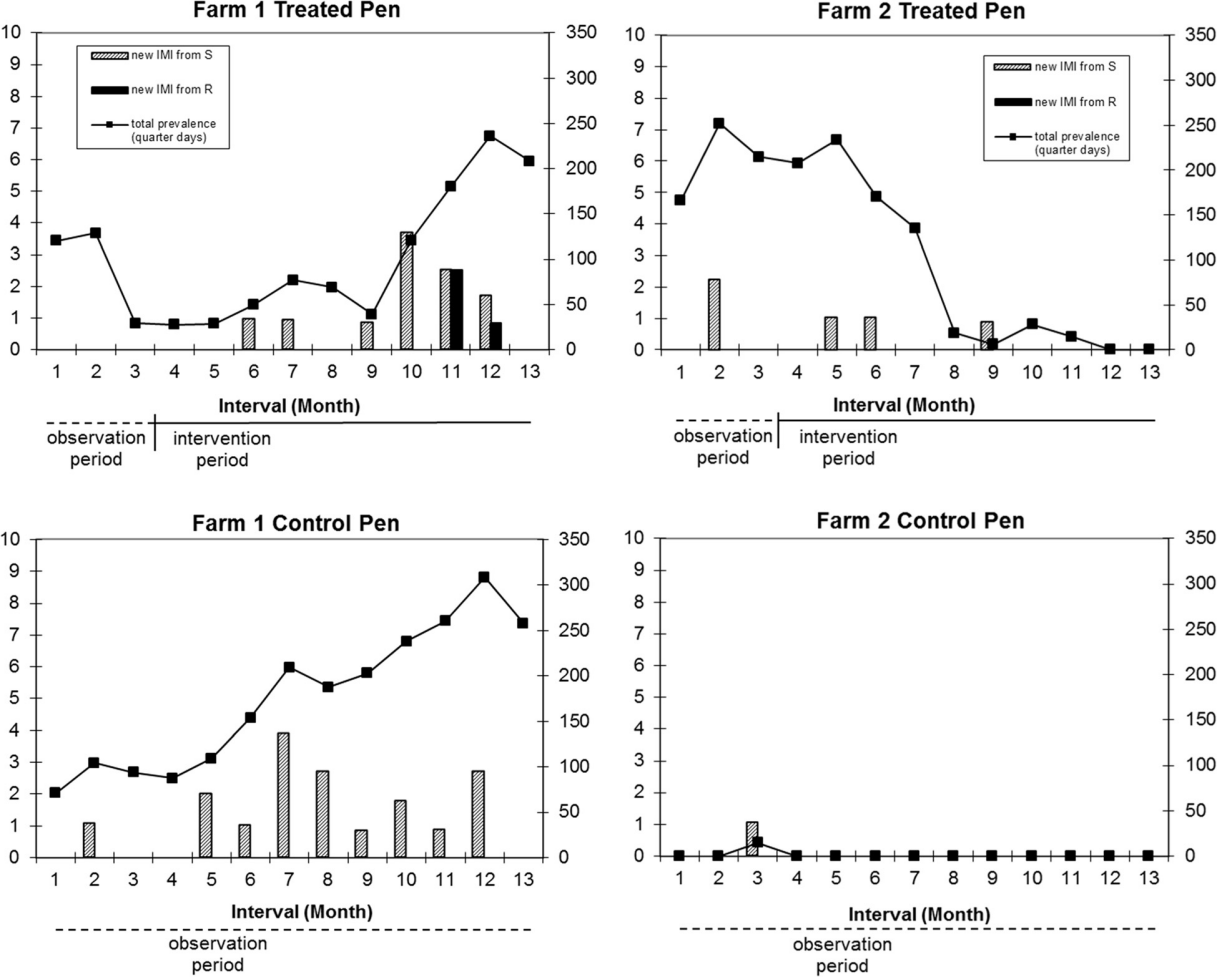
**Staphylococcus aureus intramammary infections (IMI) on two farms over 13 months. **Total prevalence (data points on line) expressed as number of days infected per 10,000 quarter days (right axis). Number new IMI from naïve susceptible quarters (**S**) and number new IMI from recovered susceptible quarters (**R**) per 10,000 quarter days at risk (left axis). In treatment pens an initial observation period of months 1-3 was followed by the intervention period during months 4-13 when cases of subclinical IMI received 8-day therapy, while control pens were observed with no intervention applied for the entire 13 months. Treatment pens top panels, Control pens bottom panels, Farm 1 left panels, Farm 2 right panels.

Differences between treatment groups were observed for the total number of infected quarter days in the two herds (Table 1). In Herd 1, the mean prevalence per 10,000 cow days for the pre-intervention time period (months 1–3) did not differ between groups; however, differences in the prevalence were evident in subsequent months in both groups (Figure 2, top panels). In the pre-intervention period (months 1–3) and the early intervention period (months 4–9) new IMIs in the treatment group were caused in approximately equal numbers by PT 1,1b, and 2, while in the late intervention period (months 10–13) subtype 2b emerged. In the control group, prevalence of PT 1 and 2 continued to rise steadily during the 13 months of the study and subtype 2b also emerged in months 10 to 13, contributing to an overall higher prevalence in this group compared to the treatment group (Figure 2).

In Herd 2, the reduction in prevalence over time was evident in the treatment group (Figure 2). This was attributed to cure of 4 IMI caused by ST8 and post-treatment spontaneous cure of an IMI caused by ST25. These cures were partially offset by a new IMI caused by ST352 in months 9 and 10 which spontaneously cured by month 12. Because there were so few *S. aureus* infections in the control pen, comparison of treatments on farm 2 was not possible, and we subsequently modeled the effect of treatment on prevalence using only the subset of data for Herd 1 in model 1.1. In the final linear model the interaction between time period and treatment group was significant, and the effect of treatment was a significant reduction in prevalence in the treated group in the early intervention period.

### Effect of lactation therapy on IMI incidence

There were a total of 42 new IMI per 501,774 quarter days at risk, with 36 new IMI observed in Herd 1 and 6 new IMI in Herd 2. When modeling incidence of new IMI in Herd 1 (model 1.2) there was no convergence in a full model due to over-parameterization of the data available for effect estimation. In a reduced model, treatment group and time period specific incidence estimates were obtained using the treatment group by time interactions and the effect of treatment was a significant reduction in incidence of new infections in the treated group in the early intervention period.

### *S. aureus* transmission parameter estimates

The transmission parameter (*β*) estimates (including 95% confidence interval lower and upper limits) for *S. aureus* from the full data set and for each individual farm were: all data, *β* = 0.00710 (0.00495 – 0.01019); farm 1, *β* = 0.00804 (0.00557 – 0.01161); farm 2 *β* = 0.00448 (0.00147 – 0.01369). Strain was the only statistically significant covariate in model 1.3. The bovine associated CC705, which includes ST151, had a significantly increased transmission parameter, [*β* = 0.01038 (0.00718 – 0.01499)] compared to the human associated CC5 [ST8; *β* = 0.00303 (0.00166 – 0.00552)], and CC97 [*β* = 0.00589 (0.00271 – 0.01280)].

## Discussion

This study demonstrated a significant increase in bacteriologic cure of *S. aureus* in quarters receiving 8-day pirlimycin therapy during lactation compared to the untreated quarters. As with previous studies of small numbers of natural infections [5] these cure proportions should be interpreted with caution. The small number of IMI caused by *S. aureus* in the two herds in our study and the high proportion of cure among treated quarters also limited our ability to explore host or strain effects associated with cure [16]. We observed a significant reduction in duration of infection in the treatment pens compared to control pens. Few mastitis control studies have included duration of infection or a survival function as an outcome. Yet duration of infection is a critical parameter in modeling indirect population level effects of interventions such as antibiotic treatment, vaccination or segregation and culling [9].

Studies designed to estimate indirect effects of interventions by quantifying species specific IMI incidence rates are uncommon as they require intensive sampling schemes [17]. To our knowledge, longitudinal studies of mastitis control with strain specific estimates of prevalence, incidence and transmission parameters have not been reported previously. Despite extensive sample collection, the current study is constrained by an issue of limited data availability with regards to *S. aureus* IMI, which might be resolved by replication on a larger number of farms. Even so, these data suggest that mastitis transmission may differ among strains of *S. aureus*. We also provide novel data demonstrating the indirect effects of lactation therapy and the potential impact of treatment-based interventions on *S. aureus* mastitis transmission dynamics.

In both herds new *S. aureus* IMI emerged in the absence of observed IMI of the same ST in other animals. We also observed that a reduction of a dominant strain was followed by emergence and possible transmission of a novel strain, and thus strain typing was sufficient to demonstrate that some new infections were not the result of contagious transmission. This suggests that in herds with a low *S. aureus* IMI prevalence, (e.g. Herd 2 in this study), a greater proportion of new IMI may be from extra-mammary sources compared to those resulting from contagious transmission.

Previous authors have demonstrated an association between non-bovine associated STs and carriage of penicillin resistance [16]. Our finding of ST8 isolates being penicillin resistant and no antimicrobial resistance among bovine-associated STs is consistent with this previous report. ST8 is a human-associated strain that has been previously reported to cause mastitis in dairy cattle in Japan, Switzerland, Turkey and the Netherlands [16,30,31]. To the best of our knowledge this is the first report of beta-lactam resistant ST8 isolated from dairy cattle in the United States. The complex relationships between host-adaptation, carriage of antimicrobial resistance and probability of cure among *S. aureus* strains warrants continued study [16].

An important observation from this study is the report of new *S. aureus* infections caused by a different strain type in previously cured quarters. In our study and that of Luby and Middleton [32], strain typing of pre- and post-treatment *S. aureus* isolates provided improved estimates of cure and re-infection proportions. In the absence of strain typing we would have under-estimated the cure proportion (67% instead of 78%) and the re-infection rate. This contrasts to a prior study where all non-cured quarters following treatment appeared to be persistently infected with the pre-treatment strain [33]. Previous studies on subclinical mastitis suggested that quarters that had recovered from *S. aureus* mastitis were more likely than naive quarters to become infected with *S. aureus*[34], and our results support this possibility. We cannot distinguish between non-cure and cure followed by re-infection with the same strain type pre- and post-treatment. This dilemma has been identified before [33], and may be due to limited strain diversity commonly observed within herds [13,35]. In addition, we cannot eliminate the possibility of genetic changes resulting in a change in PT following treatment although the criteria used for interpretation of banding patterns were developed to allow for recognition of subtypes that emerge through a genetic event [22,36]. Finally, the possibility of carriage of more than one strain and isolation of different strains pre-treatment and post-treatment has also been described [37].

Treatment of subclinical mastitis was associated with lower rates of *S. aureus* clinical mastitis which we attribute to shorter duration of infection in the treated group. A reduction in the number of clinical mastitis cases associated with lactation therapy for subclinical mastitis is in agreement with some reports [11,38], and contrasts with others [39]. Reducing the use of antibiotic treatments for clinical mastitis has been proposed to offset the costs of treating subclinical mastitis [40].

Treatment clearly eliminated infections and reduced prevalence of *S. aureus* in both herds; however the association between prevalence and incidence could only be compared between treatment and control groups in Herd 1. For this herd, we suggest that the positive association between treatment and lower IMI incidence was through the indirect effect of reduced IMI prevalence. This is consistent with a causal model for contagious disease transmission, where reducing duration of infection and overall prevalence lowers the force of infection [9]. In a transmission model Barlow et al. previously predicted that the indirect effect of therapy would approach zero under scenarios of very low transmission parameter estimates, for example in herds where post-milking hygiene is consistently applied and selective culling of chronically infected cows is implemented [9]. In this field study we observed 36 new IMI per 260,238 quarter days at risk in herd 1, and estimated the transmission parameter *β* = 0.008 and a positive indirect effect of treatment in line with our prior model predictions [9].

Transmission parameter estimates for CCs were ‘pooled’ estimates including the respective STs or PTs and PT sub-types. In the current study, as in a previous study of three herds [12], it was difficult to separate strain effects from herd effects because strains were largely herd-specific. Differences in transmission have been described for two *S. aureus* strains that occurred during the same time period within a single herd [41]. Additional research is required to quantify differences in transmission probabilities among *S. aureus* strains associated with bovine mastitis, and the potential relevance of these differences to mastitis control practices. In our data, strains belonging to the bovine adapted CC 705 appeared to have a higher transmission probability compared to a human adapted ST 8, suggesting that the greatest indirect effects of *S. aureus* control would be realized through elimination of bovine-adapted strains from herds. Because our results are based on a small number of IMI from only 2 herds further studies are needed to confirm these findings. Similar observations and recommendations have been reported for *Streptococcus agalactiae*, i.e. a differentiation between bovine and human-adapted strains, which differ in transmission probability and in the need to implement stringent control programs [42]. As a research tool, MLST is unambiguous, reproducible, portable and able to discriminate among bovine and non-bovine adapted *S. aureus* strains; however a less expensive and less time-consuming method would be required for diagnostic decision making in a clinical setting.

The treatment program used in this study was a research protocol designed to account for within cow correlation of infection risk among quarters. It is not expected that intramammary lactation treatment of all quarters in a cow with IMI in less than 4 quarters would be implemented in a commercial setting. If shown to be beneficial, lactation therapy would be integrated with other established control methods. As we have previously described, positive indirect effects of lactation therapy would be expected only in herds that have well established mastitis control practices such as post-milking hygiene and segregation or culling of chronically infected cows [9].

It is currently uncertain if lactation therapy of subclinical *S. aureus* mastitis can be economically justified. Swinkels et al. [40] have described a partial budget model for estimation of economic benefits of treating subclinical mastitis caused by *S. aureus*. Their model was sensitive to estimates of transmission probability and probability of culling. In addition, the probability of clinical mastitis was a factor contributing to the economic benefits of lactation therapy. Results of our study provide information on the indirect effects of treatment which might be used to inform additional studies on the potential cost-benefit of lactation therapy for control of subclinical mastitis in dairy herds.

## Conclusion

Treatment of subclinical *S. aureus* mastitis during lactation resulted in increased cure, reduced duration of infection, reduced rates of clinical mastitis and lower new infection rates. Therefore this is one of the first field studies to quantify positive direct and indirect effects of treating *S. aureus* mastitis during lactation. In addition strain specific transmission dynamics and transmission parameters were demonstrated. Further research on treatment of subclinical mastitis during lactation is required and future research on *S. aureus* epidemiology and control in dairy herds should include methods to improve our understanding of transmission and host-adaptation of *S. aureus* strains or clonal complexes in dairy cattle populations.

## Competing interests

YHS and Cornell University received unrestricted funding from Pfizer Animal Health to conduct a portion of this research. JWB, RNZ and YHS have received travel reimbursements from Pfizer Animal Health for presentations they have given on mastitis control in dairy herds.

## Authors’ contributions

JWB participated in study design and coordination, participated in sample collection and coordination, conducted molecular genetic testing, MLST analysis and antimicrobial susceptibility testing, conducted data management and analysis, completed statistical analysis and drafted the manuscript. RNZ participated in study design and coordination, conducted and coordinated PFGE analysis and helped to draft the manuscript. YHS conceived of the study, participated in its design and coordination, participated in statistical analysis and helped to draft the manuscript. All authors read and approved the final manuscript.
